# Is respectful care provided by community health workers associated with infant feeding practices? A cross sectional analysis from India

**DOI:** 10.1186/s12913-021-07352-w

**Published:** 2022-01-22

**Authors:** Nadia Diamond-Smith, Lakshmi Gopalakrishnan, Dilys Walker, Lia Fernald, Purnima Menon, Sumeet Patil

**Affiliations:** 1 University of California, San Francisco, 550 16th Street, 3rd Floor, San Francisco, CA 94158 USA; 2 UC Berkeley School of Public Health, 2121 Berkeley Way, Berkeley, CA 94704 USA; 3 International Food Policy Research Institute, 9th Floor, Aggarwal Corporate Tower, Govind Lal Sikka Marg, Rajendra Place, New Delhi, 110008 India; 4 Neerman, Unit 3, Mahendra Industrial Estate, Road No. 29, Sion Fort, Sion East, near VVF Ltd, Maharashtra 400022 Mumbai, India

**Keywords:** Anganwadi workers, Quality interactions, Quality of care, Infant health, Health behaviors, Nutrition

## Abstract

**Objectives:**

Breastfeeding and complementary feeding practices in India do not meet recommendations. Community health care workers (CHWs) are often the primary source of information for pregnant and postpartum women about Infant and Young Child Feeding (IYCF) practices. While existing research has evaluated the effectiveness of content and delivery of information through CHWs, little is known about the quality of the interpersonal communication (respectful care). We analyzed the effect of respectful interactions on recommended IYCF practices.

**Methods:**

We use data from evaluation of an at-scale mHealth intervention in India that serves as a job aid to the CHWs (*n* = 3266 mothers of children < 12 m from 841 villages in 2 Indian states). The binary indicator variable for respectful care is constructed using a set of 7 questions related to trust, respect, friendliness during these interactions. The binary outcomes variables are exclusive breastfeeding, timely introduction of complimentary feeding, and minimum diet diversity for infants. We also explore if most of the pathway from respectful care to improved behaviors is through better recall of messages (mediation analysis). All models controlled for socio-economic-demographic characteristics and number of interactions with the CHW.

**Results:**

About half of women reported positive, respectful interactions with CHWs. Interactions that are more respectful were associated with better recall of appropriate health messages. Interactions that are more respectful were associated with a greater likelihood of adopting all child-feeding behaviors except timely initiation of breastfeeding. After including recall in the model, the effect of respectful interactions alone reduced.

**Conclusions:**

Respectful care from CHWs appears to be significantly associated with some behaviors around infant feeding, with the primary pathway being through better recall of messages. Focusing on improving social and soft skills of CHWs that can translate into better CHW-beneficiary interactions can pay rich dividends.

**Funding:**

This study is funded by Grant No. OPP1158231 from Bill and Melinda Gates Foundation. Trial registration number: 10.1186/ISRCTN83902145

## Introduction

Community health workers (CHWs) play an essential role in many low and middle-income countries and are positioned at the frontlines to provide maternal, newborn and child health services, and promote health behaviors [1]. Evidence from low-and-middle-income countries suggests that interventions delivered by at-scale CHW programs have shown improvement in maternal, neonatal and child health outcomes [2–5]. A recent mixed-methods systematic review focused on effects of CHW programs on inequities from intervention coverage to behavioral and mortality outcomes across continuum of care suggests that in some contexts, interventions involving CHW home visits and community-based group interventions have had moderate success in reducing inequities in maternal and new-born health intervention coverage and behaviors [6]. Strengthening CHWs programs can leverage these successes and have been recognized as essential to reach every community and household to achieving universal coverage of key community-based evidence–based interventions and health services by 2030 [1].

India has strong government-led national Community Health Worker (CHW) programs with three cadres of over 2 million all-female CHWs delivering services at the frontlines. Accredited Social Health Activists (ASHAs) under National Health Mission and the Anganwadi Workers (AWWs) under the Integrated Child Development Services (ICDS) generally work together to serve pregnant, lactating women, and infants at the village-level serving a catchment area of 800–1000 individuals [7–9]. Several studies from India have highlighted the positive role played by community health worker (CHW) programs on a range of reproductive, maternal, and neonatal health including promotion of reproductive health and contraceptive services, birth preparedness, antenatal care during pregnancy, skilled birth attendance during delivery, facility delivery, immunization coverage, and neonatal and infant mortality [10–15]. Few studies have demonstrated usefulness of CHW interventions in bridging inequities in maternal and neonatal health behaviors [6]. For instance, a study in Bihar found that households belonging to a lower socio-economic status had greater odds of receiving food supplementation compared with households in the highest socio-economic status [7]. Another study from rural Uttar Pradesh found CHW’s services for birth registration to be greater among women with higher socio-economic class and education compared to lower socio-economic class and education [16]. A review of interventions in India that promoted various parts of infant and young child feeding found that interventions that used community health workers to promote initiation of breastfeeding and feeding frequency were generally successful [17].

A growing body of evidence recognizes the role of quantity of home visits by CHWs on pregnancy care and maternal and newborn care behaviors in South Asia including India. A systematic review and meta-analysis of home visits by CHWs to prevent neonatal death in resource-poor settings with poor access to facility-based care concluded that antenatal and neonatal care home visits by CHWs were associated with lower neonatal mortality and stillbirths in South Asian settings [18]. A large-scale cluster randomized cluster-controlled trial in Haryana (India) studying the impact of postnatal home visits by CHWs demonstrated that home visits by CHWs during the postnatal period was associated with lower infant and neonatal mortality rate, with substantial reductions observed in home-based births compared to facility-based births [12]. A recent study from Uttar Pradesh in India found that when pregnant women received multiple home visits by CHWs (ASHAs) including at least one visit early in their pregnancy, they were more likely to attend antenatal check-ups, consume iron and folic acid (IFA), and deliver at a health facility. CHW’s presence during childbirths was also associated with greater early initiation of breast feeding and respectful maternity care from health facility staff. Further, receiving one or more visits from CHWs in the first week of birth (compared to none) was associated with higher likelihood of exclusive breastfeeding and clean cord care [19]. All the studies examined focus on life-stage appropriate quantity of visits, but to our knowledge, we found scant evidence on how quality of CHW home visits impact maternal and newborn care behaviors.

The quality of CHW care provision has received considerably limited attention from researchers. Some frameworks have considered factors that influence effectiveness of CHWs, including aspects of quality, but much of the focus has been on systems level factors [20, 21]. Less attention has been paid to what happens when the CHW is interacting with a beneficiary/client, and how that interaction is associated with care-seeking behaviors. Respectful care, also called person-centered, interpersonal, woman-centered care, or described as part of the experience of care, is receiving increasing attention globally across the peripartum period as an important domain of quality. The WHO quality of care framework for maternal and newborn health specifically highlights experiences of care as a key component of quality [22]. This domain of quality of care includes domains related to the interaction between the health care provider and the client (woman), including respect, communication, trust, etc. [23]. Respectful maternity care has been found to be associated with improved care-seeking and maternal health outcomes [24, 25], and respectful family planning care is associated with family planning knowledge, method uptake and continuation [26, 27].

The majority of the research on respectful care has focused on interactions that occur within health facilities, which mirrors the fact that most of this research has also been focused on the time around childbirth. There is a substantial body of literature documenting poor person-centered or disrespectful experiences that women face at the time of delivery, globally, including in India [28–31]. However, there is much less evidence about person-centered interactions between women (clients) and CHWs, even though, as discussed above, CHWs are often the first and primary source of contact and information for many women globally, throughout pregnancy and postpartum.

Few studies have specifically looked at respectful care and CHWs, despite the fact that part of the rationale for engaging community members to provide care was that they would have community buy-in, trust, understand the cultural context, and be best able to communicate with community members [32]. Some studies of CHWs explore aspects of respectful care as one part of a broader focus on quality, for example, a qualitative study in Bangladesh about the quality of services by CHWs for malnutrition explored “acceptability” of the CHWs, found them acceptable and valuable to the community [33]. Taking this one step further, little is known about how respectful (or disrespectful) care provided by CHW is associated with health outcomes. A study with CHWs also providing family planning in India found that higher person-centered care was associated with method continuation [27]. A few studies have specifically looked at domains of respectful care and CHWs, most often trust. A qualitative study on CHWs in South Africa providing maternal and child health services found that lack of trust and concerns over confidentiality were barriers to care provision [34]. Two qualitative studies from India exploring experiences of CHWs in strengthening maternal health services found that CHWs were unable to inspire trust and credibility in their communities, which was thought to be because of limited community involvement in selection of CHWs and lack of timely receipt of payments linked to government conditional cash transfer programs [35, 36].

India has 1.4 million Anganwadi Workers (henceforth referred to as AWWs) who provide health and nutrition-related services to pregnant and postpartum women as part of the national flagship program, Integrated Child Development Services (ICDS). AWWs work at Anganwadi Centers (AWCs), early childhood development and feeding centers at the village-level that caters to a catchment area of 800–1000 individuals. AWWs, along with another cadre of CHWs called Accredited Social Health Activists (ASHAs), are the primary contact and main source of information to pregnant and postpartum women in India. Along with counselling and information on topics such as breastfeeding, pregnancy and postpartum complications, contraception, and more, these CHWs are also tasked with linking women with health services (for example, accompanying them to the facility for childbirth). Specifically related to nutrition, AWWs deliver five essential services for the nutrition program including: supplementary food, home visits to inform pregnant and lactating women on pregnancy care and infant and young child feeding practices growth monitoring of children, pre-school education activities, and organize a monthly fixed-day event—village health and nutrition days (VHND) for immunization and other health-related services [7]. AWWs are part-time, female workers receiving an average monthly fixed honorarium of about USD 60 (INR 4500), although there is variation in honorarium across the country [7].

A recent paper on ICDS coverage by Chakrabarti and colleagues highlight an increase in usage of ICDS services between 2006 and 2016 (9.6 to 37.9% for supplementary food, 3.2 to 21.0% for health and nutrition education, 4.5 to 28% for health check-ups and 10.4 to 24.2% for child-specific services (e.g. immunization, growth monitoring), however, they noted the program’s failure to reach the households from the lowest socioeconomic strata and women with low schooling levels especially in states with the highest burden of undernutrition ([37]. Both Madhya Pradesh (MP) and Bihar (the focal states in our study) have a high burden of undernutrition indicated by high under-five mortality of 65 and 58 per 1000 live births, stunting (43.6% of children aged 0–5 years in MP, and 49.3% in Bihar), and more than 55% of anemic pregnant women respectively. Compared to MP, Bihar has worse indicators on ICDS coverage and services – 49% children below the age of 6 received any service from an AWC, 50% of mothers who were weighed received weighed at an AWC received counselling from an AWW, 35% of mothers with children below the age of 6 years received any service from an AWC during pregnancy and lactation in Bihar. MP was much better in ICDS coverage as well as services - 63% children below the age of 6 received any service from an AWC, 62% of mothers who were weighed at an AWC received counselling from a AWW, 71% of mothers with children below the age of 6 years received any service from an AWC during pregnancy and lactation. Further, only 34% of infants in Bihar and MP were fed within the first hour of birth, and exclusive breastfeeding at 6 months was 53 and 58%, respectively. Even more alarmingly, only 8% in Bihar and 7% in MP of children 6–23 months received an adequate diet [38]. These staggering figures are not entirely surprising since past evaluations of ICDS found gaps in several operational, infrastructure, and service delivery-related deficiencies including poor record-keeping, ineffective monitoring of services, low quality supplementary food, increased burden on AWWs duties, and dilution of focus or competing priorities with undernutrition-related work [39–43].

The first 1000 days of life, that includes maternal nutrition during pregnancy and child nutrition during the first 2 years of life are critical periods of growth and neurodevelopment in a child’s life with intergenerational consequences [44]. Appropriate infant and child feeding practices (IYCF), which include early initiation of breastfeeding, exclusive breastfeeding until 6 months and provision of complementary foods along with breastfeeding from 6 months to 23 months of age, are a crucial part of optimal growth and development [45]. CHWs are the frontline workforce that link communities to formal health systems and educate women and children on topics related to nutrition and encouraging mothers to take-up infant and child feeding practices in their catchment areas. Thus, understanding the relationship between respectful care provision by CHWs and uptake of nutritional behaviors among women can provide evidence on one of the ways to strengthen maternal knowledge and IYCF practices.

**Fig. 1:**
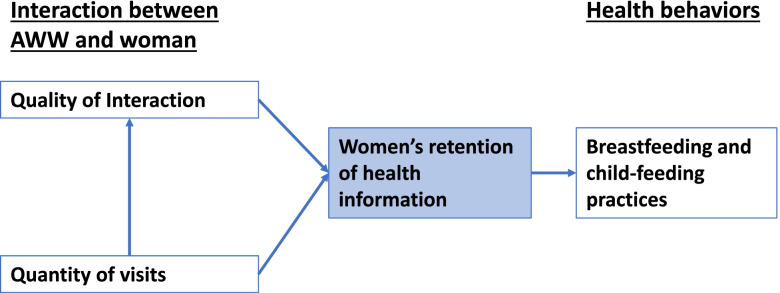
Conceptual and Analytical Model

Based on the previous studies described above, we hypothesize that respectful interactions between AWWs and pregnant and postpartum women will be associated with better adherence to the recommended IYCF guidelines, specifically through the pathway of improved knowledge of appropriate practices and health information (Fig. 1). Other factors unrelated to the person-centeredness of the interaction might both affect how a woman rates the person-centeredness of the interaction, and her health behaviors, specifically the number of visits by the AWW. We hypothesize that a greater number of visits would increase both the level of respectful care and improve infant feeding behaviors. The socio-demographic characteristics of women and their household characteristics will also need to be adjusted for, as they might impact how information and the experience is translated into feeding behaviors.

## Methods

### Data

We use data from a larger impact evaluation designed to test the effectiveness of a mobile technology-based intervention for AWWs. The technology for the intervention was aimed to support the AWWs with tracking and managing beneficiaries, recording data, scheduler to prompt them to undertake timely home visits, and had some behavior-change communication videos. By controlling for the number of home visits made by CHWs, we adequately address the key element the technology could influence that could also potentially be associated with the outcomes. The larger parent study included both a baseline and end-line survey, and we only use the end-line survey data for this analysis, since it is not reporting on an outcome related to the intervention. The end-line survey was conducted by a survey team appointed by Network for Engineering and Economics Research and Management in 2019 across 12 districts in the two north Indian states of Madhya Pradesh (MP) and Bihar [46]. Using propensity score matching, 852 villages were selected from these 12 districts and then up to two AWCs were sampled per village and up to eight mothers of children < 12 m and up to three pregnant women in their last trimester were randomly sampled based on the AWW registries. All study participants provided verbal informed consent before data collection and were surveyed using structured computer-assisted personal interviews. Surveys were conducted by a female interviewer who spoke the local language in the respondent’s preferred location, usually the respondent’s home, at a time that was most convenient for the respondent. Surveys covered topics related to knowledge and services use for pregnancy, delivery, postpartum nutrition and health services. A total of 5679 women were surveyed, and of those 1753 had children < 6 months and 1513 had children 6–12 months. Study protocols were reviewed and approved by institutional review boards at the University of California, Berkeley (Ref. No. 2016-08-9092), and the India-based Suraksha Independent Ethics Committee (Protocol No. 2016-08-9092).

#### Socio-demographics variables

We include the following socio-demographic variables in our models: age in years (continuous), years of education (continuous), women’s work outside the home (yes/no), total number of pregnancies (continuous), caste (belonging to low caste (scheduled caste or tribe) compared to higher caste/no caste), religion (Hindu compared to other), and wealth (quartiles). We also include an indicator variable for the state and arm of the intervention study.

#### Quality (respectful care)

To measure the interaction between AWWs and women, we collected seven questions related to the woman’s experience of receiving care/counselling from the AWW during home visits or contact at the Anganwadi Center. Specifically, we asked if the woman felt that she could trust the AWW, the AWW had her best interest in mind, was interested in her health, treated her with respect, spent time with her, talked in a friendly manner, and that she felt comfortable asking the AWW questions. A binary variable was made for women who reported positively on all questions (1) compared to women who only reported positively on 6 or fewer (0).

#### Quantity

We include a variable for women receiving the adequate number of home visits from the AWW based on her postpartum stage. This was included to account for any additional impact of the mobile technology (which was not the main focus of this study), aside from simply controlling for the technology intervention itself.

#### Knowledge

Women were asked a series of questions about specific messages that the AWW was supposed to have provided to her. To measure knowledge, we created a variable indicating if a woman remembered at least 50% of these messages. Women received different messages based on her specific point of postpartum, and by structuring the variable in this way, it is comparable across postpartum stages.

#### Primary behavioral outcomes

We explore the impact of quality of care on five infant feeding behavioral outcomes:

1. Exclusive breastfeeding: Proportion of women with infants 0- < 6 months old who report feeding their child only breastmilk in the 24 h preceding the survey.
2. Breastfeeding initiation: Proportion of women with infants 0–12 months who reported feeding their infant within the first hour of birth.
3. Adequate diet: Proportion of infants who received an adequate diet per their age, in the 24 h preceding the survey.
4. Adequate number of meals: Proportion of infants 6–12 months who received the adequate number of meals in the 24 h preceding the survey.
5. Dietary diversity: Proportion of infants 6–12 months who received at least 4 or more food groups in the 24 h preceding the survey.

First, we describe the socio-demographics of our population, and women’s interaction with the AWW, including the respectful care measures, using frequencies and means. We then show the proportion of women reporting each infant feeding behavioral outcome separated out by women who had a high compared to a low respectful care score. We look for differences by sub-group using chi squared tests. Next, we conduct a mediation analysis to understand if the pathway from respectful care to behavior change acts through knowledge, using the Baron and Kenny (1986) method [47]. To do this, we first explore the association between respectful care and knowledge of health behaviors. Then, we run a series of 5 multi-variable logistic regression models exploring the association between respectful care and each of the five infant feeding behavioral outcomes, first with and then without the knowledge variable, and then adjusting for the other quality and socio-demographic variables. All models accounted for the survey study design and were clustered at the block level. Data was analyzed using STATA v 15.

## Results

Women in our sample were on average 24 years old, had 4.8 years of schooling, and were Hindu (92.3%) (Table 1). Almost half (44.3%) were scheduled caste or tribe, and 17.3% were in paid work. About a third, 35.87%, received the adequate number of visits from the AWW, as defined by the government program based on their postpartum stage. About a quarter (23.5%) of women could remember at least 50% of the appropriate messages given their postpartum stage.

**Table 1:** Socio-demographics of the study population [*n* = 5679]

	n	%
Age (mean, IQR)	24.23	21–26
Number of pregnancies (mean, IQR)	2.6	1–3
Education in years (mean, IQR)	4.8	0–9
Paid Work	1564	17.3
Scheduled Caste or Tribe	4006	44.3
Hindu	8366	92.3
Recalls at least 50% of counseling messages	1559	23.5
State		
Bihar	4767	52.8
Madhya Pradesh	4266	47.2
Received adequate number of visits from AWW	2380	35.87

Overall, most women reported their interaction with AWWs highly, with 68–76% of women reporting positive answers for respectful care items (Table 2). Lowest ranking items included: feeling that the AWW spent time at the visit (68%) and that they felt comfortable asking questions (69.8%). Highest ranking included feeling that the AWW treated them with respect (76.8%) and talked in a friendly manner (76.8%). Overall, just over half (54%) of women reported positive rankings for every single respectful care indicator.

**Table 2:** Components of respectful interaction between women and AWWs

	No.	%
Felt like you could trust the AWW		
No	1357	23.9
Yes	4322	76.1
Felt that the AWW had your best interest in mind		
No	1532	27.0
Yes	4147	73.0
Felt the AWW was interested in your health		
No	1532	27.0
Yes	4147	73.0
Felt the AWW treated you with respect		
No	1320	23.2
Yes	4359	76.8
Felt the AWW spent time at your visit		
No	1820	32.0
Yes	3859	68.0
Felt the AWW talked in a friendly manner		
No	1319	23.2
Yes	4360	76.8
Felt comfortable asking the AWW questions		
No	1714	30.2
Yes	3965	69.8
Binary Summary Score: 1 = positive on all indicators, 0 = positive on 6 or less		
Not positive	2612	46.0
Positive	3067	54.0
Total	5679	100.0

Most (70.3%) of the women with infants under 6 months fed their infants only breastmilk on the previous day, with significantly more women with a respectful interaction with the AWW (76.6%) compared to those without (60%) reporting doing so (Table 3). Most (84.1%) of the women with children 0–12 months breastfed their infants within an hour of birth, again with significantly more women with positive relationships (85.1%) doing so compared to those without (82.4%). Less than half (43.3%) children 0–12 received an adequate diet in the last 24 h based on their age, with more of those with a positive relationship with the AWW (47.5%) doing so compared to those without (37.3%). Just over half (58%) children 6–12 months received the adequate number of meals in the last 24 h, with no significant difference by group. Finally, only 15% of children 6–12 months received 4 or more food groups the previous day, with significantly more with a positive relationship (16.7%) compared to those without (12%) doing so.

**Table 3:** Bivariate association between child feeding indicators and respectful care (binary)

	Low respectful care		High respectful care		Total	
	No.	%	No.	%	No.	%
% infants 0- < 6 m of age who received only breastmilk during the previous day***						
0	267	40	257	23.4	524	29.7
1	400	60	841	76.6	1241	70.3
Total	667	100	1098	100	1765	100
% beneficiaries with 0-12 m child who breastfed their child within an hour of birth*						
0	217	17.6	306	14.9	523	15.9
1	1015	82.4	1743	85.1	2758	84.1
Total	1232	100	2049	100	3281	100
Children aged 0-12 m who received adequate diet as per their age in last 24 h***						
0	773	62.7	1076	52.5	1849	56.4
1	459	37.3	973	47.5	1432	43.6
Total	1232	100	2049	100	3281	100
Children aged 6–12 months who received adequate number of meals in last 24 h						
0	254	45	382	40.2	636	42
1	311	55	569	59.8	880	58
Total	565	100	951	100	1516	100
Children 6–12 months received 4 or more food groups*						
0	497	88	792	83.3	1289	85
1	68	12	159	16.7	227	15
Total	565	100	951	100	1516	100

p* < 0.5, p** < 0.01, p*** < 0.001

More respectful interactions are associated with increased recall of messages (OR = 2.49, 95% CI: 1.98–3.15) (Table 4). More respectful interactions are also associated with exclusive breastfeeding in the previous day (OR = 1.55, 95% CI: 1.22–1.98), but not breastfeeding initiation. After adding recall of messages to the model, interactions that are more respectful are still associated with exclusive breastfeeding, as is recall. Women’s education increases the likelihood of exclusive breastfeeding, and higher wealth decreases the likelihood of both breastfeeding behaviors. Receiving the adequate number of visits is associated with decreased likelihood of breastfeeding exclusivity, but increased likelihood of breastfeeding initiation. Being in the intervention (treatment) arm was also associated with message recall (OR = 1.6, 95% CI: 1.34–1.92).

**Table 4:** Association between respectful care, knowledge recall and breastfeeding practices, multivariable logistic regression models (*Odds Ratios, 95%CI)*

	Recall 50% of messages	Only breastmilk last 24 h		Breastfed within 1 h of birth	
Population	Children 0–12 months	Children< 6 months		Children 0–12 months	
More respectful interaction with AWW (binary)	2.49*** (1.98–3.15)	1.55*** (1.22–1.98)	1.40*** (1.09–1.79)	1.12 (0.87–1.44)	1.02 (0.79–1.32)
Recall 50% of messages			1.60*** (1.22–2.08)		1.58*** (1.29–1.94)
Woman’s age (in years)	1.00 (0.98–1.02)	0.99 (0.95–1.03)	0.99 (0.95–1.04)	0.99 (0.96–1.03)	0.99 (0.96–1.03)
Woman’s education (in years)	1.02** (1.00–1.04)	1.04** (1.01–1.07)	1.04** (1.01–1.07)	1.01 (0.99–1.04)	1.01 (0.98–1.04)
Woman working outside the house (compared to not)	1.12 (0.89–1.39)	0.88 (0.59–1.29)	0.87 (0.58–1.30)	0.67*** (0.51–0.89)	0.66*** (0.50–0.87)
Parity	1.02 (0.96–1.07)	0.99 (0.90–1.09)	0.99 (0.90–1.09)	1.09 (0.97–1.21)	1.08 (0.97–1.21)
Scheduled Caste or Tribe (compared to no/higher caste)	0.74*** (0.63–0.88)	1.25* (0.99–1.57)	1.30** (1.03–1.64)	0.92 (0.70–1.22)	0.95 (0.72–1.25)
Hindu (compared to not Hindu)	1.25 (0.87–1.79)	1.45* (0.93–2.24)	1.40 (0.91–2.16)	1.40 (0.86–2.27)	1.37 (0.84–2.22)
Wealth quartile	1.08* (1.00–1.17)	0.88* (0.77–1.01)	0.87** (0.76–0.99)	0.86*** (0.78–0.95)	0.85*** (0.77–0.94)
Received adequate number of visits	1.50*** (1.26–1.78)	0.46*** (0.32–0.66)	0.43*** (0.30–0.61)	1.39*** (1.17–1.66)	1.33*** (1.12–1.59)
State	1.04*** (1.02–1.05)	1.17*** (1.15–1.20)	1.17*** (1.14–1.19)	1.03*** (1.01–1.06)	1.03** (1.01–1.06)
Treatment Arm	1.60*** (1.34–1.92)	1.19 (0.92–1.54)	1.12 (0.85–1.47)	1.02 (0.78–1.33)	0.97 (0.74–1.27)
Constant	0.08*** (0.04–0.16)	0.13*** (0.05–0.35)	0.14*** (0.05–0.35)	2.38* (0.92–6.13)	2.48* (0.96–6.42)
N	3266	1753	1753	3266	3266

*** *p* < 0.01, ** *p* < 0.05, * *p* < 0.1 and robust confidence interval in parentheses

Having a more respectful interaction with the AWW is associated with women being more likely to provide their infants adequate diet per their age, provide adequate number of meals and provide 4 or more food groups (OR = 1.34, 95% CI: 1.16–1.56; OR = 1.3, 95% CI: 1.01–1.67; OR = 1.55, 95% CI: 1.14–2.09, respectively) (Table 5). When we include recall in the model, we can see that most of the pathway between respectful interactions and behaviors is through improved recall of messages. Maternal education is associated with increased likelihood of adequate diet and frequency but not diversity, and having the appropriate number of visits per postpartum stage is associated with decreased likelihood of these two behaviors. Being in the treatment arm was not associated with any of the outcomes of interest.

**Table 5:** Association between respectful care, knowledge recall and child feeding practices, multivariable logistic regression models (Odds Ratios, 95%CI; p < 0.1* p < 0.05** p < 0.001***)

	Received adequate diet as per age in last 24 h		Received adequate number of meals in last 24 h		Received 4 or more food groups	
Population	Children 0–12 months		Children 6–12 months		Children 6–12 months	
More respectful interaction with AWW (binary)	1.34*** (1.16–1.56)	1.14* (0.98–1.33)	1.30** (1.01–1.67)	1.25* (0.96–1.62)	1.55*** (1.14–2.09)	1.26 (0.89–1.77)
Recall 50% of messages		2.38*** (2.07–2.75)		1.25* (1.00–1.57)		2.42*** (1.58–3.69)
Woman’s age (in years)	0.98* (0.95–1.00)	0.98* (0.95–1.00)	1.03 (0.99–1.06)	1.03 (0.99–1.06)	1.01 (0.97–1.05)	1.01 (0.96–1.05)
Woman’s education (in years)	1.03*** (1.01–1.05)	1.03*** (1.01–1.05)	1.05*** (1.02–1.08)	1.05*** (1.02–1.08)	1.02 (0.98–1.06)	1.02 (0.98–1.06)
Woman working outside the house (compared to not)	0.90 (0.74–1.11)	0.88 (0.72–1.08)	1.33 (0.93–1.90)	1.32 (0.92–1.89)	1.18 (0.75–1.87)	1.13 (0.74–1.73)
Parity	1.04 (0.98–1.12)	1.04 (0.97–1.11)	0.97 (0.90–1.04)	0.97 (0.90–1.04)	0.95 (0.84–1.08)	0.95 (0.83–1.08)
Scheduled Caste or Tribe (compared to no caste)	1.04 (0.88–1.24)	1.11 (0.92–1.34)	1.23* (0.97–1.57)	1.25* (0.98–1.59)	0.70** (0.51–0.96)	0.73* (0.52–1.01)
Hindu (compared to not Hindu)	1.27 (0.89–1.81)	1.23 (0.90–1.68)	1.33 (0.89–1.98)	1.33 (0.90–1.98)	0.62* (0.36–1.07)	0.60* (0.35–1.04)
Wealth quartile	0.96 (0.89–1.05)	0.95 (0.87–1.03)	1.12** (1.00–1.25)	1.12** (1.00–1.25)	1.13 (0.96–1.34)	1.14 (0.96–1.35)
Received adequate number of visits	0.30*** (0.24–0.36)	0.26*** (0.22–0.31)	0.54*** (0.40–0.73)	0.51*** (0.38–0.70)	1.32 (0.86–2.04)	1.10 (0.71–1.70)
State	1.10*** (1.08–1.12)	1.09*** (1.08–1.11)	0.97* (0.95–1.00)	0.97* (0.95–1.00)	0.95*** (0.92–0.98)	0.95*** (0.92–0.98)
Treatment Arm	1.20 (0.95–1.52)	1.10 (0.86–1.40)	0.94 (0.67–1.31)	0.92 (0.66–1.28)	1.26 (0.89–1.80)	1.17 (0.81–1.69)
Constant	0.27*** (0.13–0.55)	0.28*** (0.14–0.57)	0.74 (0.23–2.32)	0.74 (0.24–2.34)	0.21*** (0.07–0.64)	0.22*** (0.07–0.69)
N	3266	3266	1513	1513	1513	1513

*** *p* < 0.01, ** *p* < 0.05, * *p* < 0.1 and robust confidence interval in parentheses

### Sensitivity analysis

As a sensitivity analysis to ensure that other factors that could influence the relationship between the woman and AWW, such as caste, were not driving the association between person centered quality and behaviors, we re-ran all models with the caste of the AWW included. These models did not differ in terms of magnitude or significance (results not shown).

## Discussion

Having positive interactions with their community health workers (for example, who trust their CHW, feel respected, like they can ask questions, like the provider cares about them), is associated with women being more likely to meet some components of appropriate child feeding practices that CHWs are aiming to improve. The primary pathway through which this association occurs is by better retention of women’s knowledge (recall of messages). This suggests that supporting CHWs to be able to provide respectful care in their communities, through training and supportive supervision, and potentially lower workload and more time to be able to spend with women, could help improve maternal behaviors and subsequent infant nutritional and growth outcomes.

Timely initiation of breastmilk is the only outcome explored that does not seem to be associated with the quality of respectful care. This could be because this practice is more deeply rooted in cultural practices and beliefs than the other health behaviors. Delayed introduction of breastmilk is practiced in some parts of India because of the belief that the mothers’ milk is not ready yet [48, 49]. Counselling that directly address some of these cultural beliefs may be necessary in addition to respectful care. It is also possible that some women had a c-section, which may have delayed breastfeeding initiation but was not included in our analysis.

We hypothesized that number of times that a CHW visits a woman could be an indicator of better quality, or at least influence women’s perceptions of quality, and also, influence her recall of messages. Interestingly, while women receiving the adequate number of visits was associated with increased recall of messages, it was not consistently associated with appropriate behaviors, and in some cases, associated with women being less likely to practice those behaviors. It is possible that AWW visit women differentially—maybe focusing their efforts on women who they see being more in need of help, but who are less likely to practice certain behaviors. Regardless, understanding the relationship between number of visits and outcomes deserve more exploration.

This analysis suggests that respectful care matters for the translation of some CHW messaging into behavior change, and more research is needed to understand what other factors in combination with respectful care are most successful. Few interventions have specifically trained CHWs on domains related to respectful care. One study in Nepal which conducted training on interpersonal communication to CHWs providing family planning found evidence of improved communication, but that this was not the main barrier to women using family planning [50]. Supportive supervision of CHWs has been found to help them gain confidence and subsequently build trust with community members, but we do not know how this impacted health behaviors or outcomes [51].

This study has several strengths, notably, its large sample size of randomly selected women and the collection of data on an understudied component of quality of CHW care-- respectful care. However, there are several limitations. The first is that this is a cross-sectional study, so we are unable to make clear causal linkages. Despite the fact that the interaction with CHWs happened before the behavior, some types of women may be more likely to have or perceive positive interactions, and these women may also be more likely to adopt health behaviors. Given the retrospective nature of the data, there may be some recall bias, although women were asked questions relevant to their specific postpartum stage, so this should be minimal. Finally, we were unable to objectively measure other components of quality related to the interaction (for example, what messages the CHW told the woman) or have an objective measure of respectful care. However, the woman’s experience of the interaction is probably a more meaningful measure.

## Conclusions

CHWs are the first point of contact and main source of information for many people in LMICs, especially pregnant and postpartum women, living in rural areas who are disadvantaged in other ways. CHWs serving pregnant and postpartum women in India provide care that is respectful, caring, and that women feel positively about, and, importantly, this is associated with uptake of appropriate health behaviors. However, CHWs often face many challenges, such as lack of support, resources, training, little or delayed pay, etc. Strengthening CHW’s ability to provide respectful care could help retention of health knowledge and outcomes among women and is an essential component of providing high quality care.

## Data Availability

Availability of data and material statement: The datasets generated and/or analysed during the current study are not publicly available because our impact manuscript from our study is not yet published. All data will be made available publicly but can be available from the corresponding author on reasonable request until then. Please contact the corresponding author, Dr. Nadia Diamond-Smith (nadia.diamond-smith@ucsf.edu to request for data.
